# An in silico argument for mitochondrial microRNA as a determinant of primary non function in liver transplantation

**DOI:** 10.1038/s41598-018-21091-9

**Published:** 2018-02-15

**Authors:** Shirin Elizabeth Khorsandi, Siamak Salehi, Miriam Cortes, Hector Vilca-Melendez, Krishna Menon, Parthi Srinivasan, Andreas Prachalias, Wayel Jassem, Nigel Heaton

**Affiliations:** Institute of Liver Studies, King’s College Hospital, London, United Kingdom

## Abstract

Mitochondria have their own genomic, transcriptomic and proteomic machinery but are unable to be autonomous, needing both nuclear and mitochondrial genomes. The aim of this work was to use computational biology to explore the involvement of Mitochondrial microRNAs (MitomiRs) and their interactions with the mitochondrial proteome in a clinical model of primary non function (PNF) of the donor after cardiac death (DCD) liver. Archival array data on the differential expression of miRNA in DCD PNF was re-analyzed using a number of publically available computational algorithms. 10 MitomiRs were identified of importance in DCD PNF, 7 with predicted interaction of their seed sequence with the mitochondrial transcriptome that included both coding, and non coding areas of the hypervariability region 1 (HVR1) and control region. Considering miRNA regulation of the nuclear encoded mitochondrial proteome, 7 hypothetical small proteins were identified with homolog function that ranged from co-factor for formation of ATP Synthase, REDOX balance and an importin/exportin protein. In silico, unconventional seed interactions, both non canonical and alternative seed sites, appear to be of greater importance in MitomiR regulation of the mitochondrial genome. Additionally, a number of novel small proteins of relevance in transplantation have been identified which need further characterization.

## Introduction

Cold preservation has been the mainstay of donor liver maintenance in transplantation for the past 50 years. However, a period of cold ischemia followed by reperfusion produces an ischemia reperfusion injury that is the main determinant of graft function and outcome^1^. To ameliorate ischemia reperfusion injury and the risk of primary non function (PNF) or dysfunction, cold ischemic times are ideally kept to a minimum. Relatively little is known about mitochondrial behaviour on reperfusion, but it is recognized that the integrity and resilience of mitochondria on reperfusion is a key determinant of the extent of injury and outcome.

Mitochondria are central to cellular energy generation and sit at the crossroads of a number of critical cell pathways e.g glycolysis/OXPHOS balance, senescence and apoptosis. Mitochondria have their own genomic, transcriptomic and proteomic machinery but are unable to be autonomous, needing both the nuclear and mitochondrial genomes to coordinate the expression of at least 2000 mass spectrometry identified mitochondrial proteins, of which 13 are encoded by the mitochondrial genome (mt genome). It is estimated that only 3% of the mitochondrial proteome (mt proteome) is involved in ATP production^2,3^.

Mitochondrial microRNA (MitomiRs) are a microRNA (miRNA) species that have been characterized to localize to mitochondria. The majority of MitomiRs originate from the nuclear genome and are imported into the organelle^4,5^. MitomiR function is presently being determined but it is thought to encompass metabolic control through their ability to adjust the composition of the mitochondrial proteome and potentially, provide a language for anterograde retrograde nuclear mitochondrial communication. Our previous work on miRNA expression in PNF demonstrated miR-22 to be of importance and with predicted influence on metabolic and apoptotic pathways, both of which centre on mitochondria^6^. This observation and the continuing paucity of data on PNF has motivated the present re-analysis. Using computational biology we have explored MitomiRs and miRNA interactions with the mitochondrial proteome in the clinical transplant model of PNF in the donor after cardiac death (DCD) liver.

## Materials and Methods

### miRNA Array source data and overview of computational biological strategy

**Figure 1:**
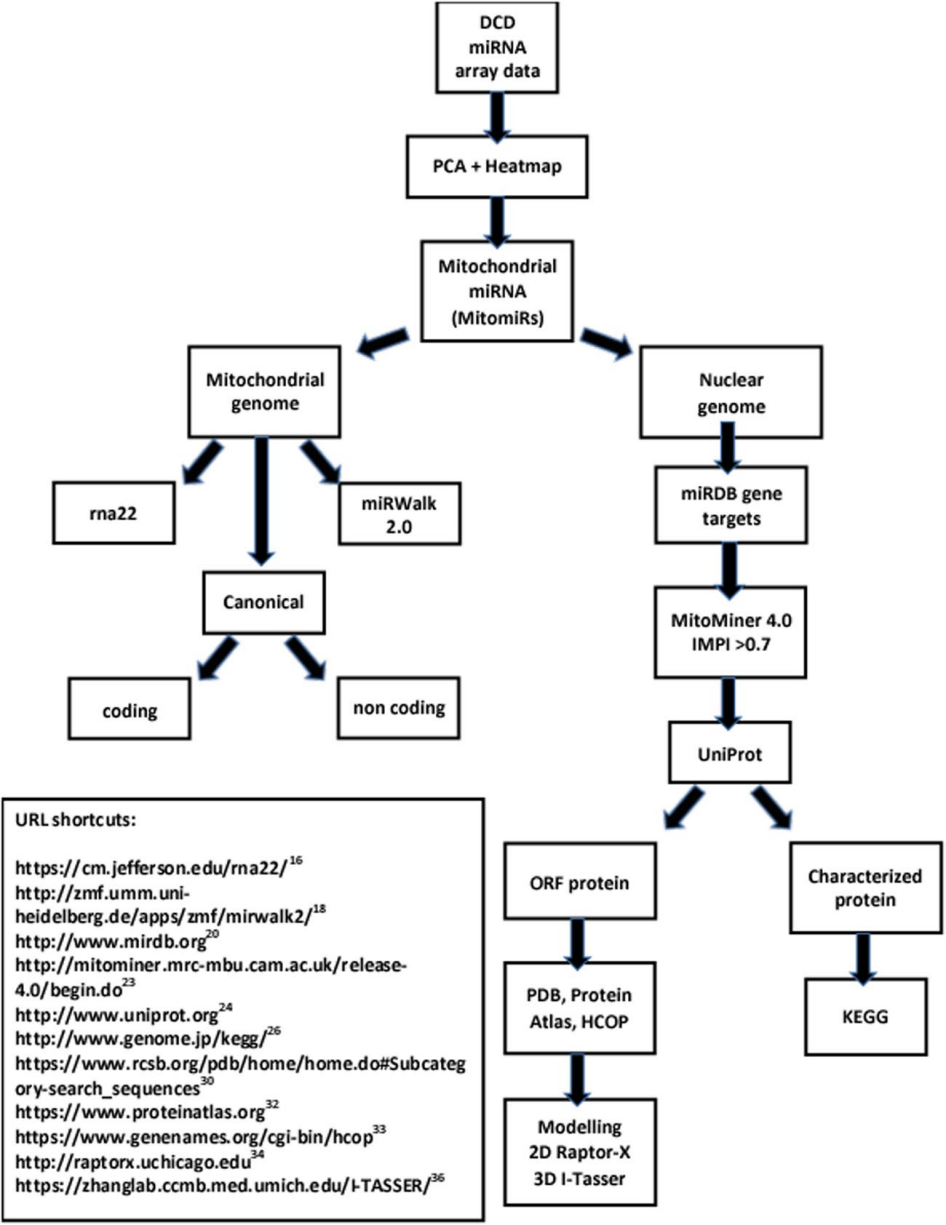
Computational work stream summarizing in silico analysis of mitochondrial microRNAs (MitomiRs) interactions with the mitochondrial and nuclear genomes. Archival miRNA array data from the donor after cardiac death (DCD) liver was filtered to identify MitomiRs. MitomiR seed interactions with the mitochondrial genome were explored by applying simple canonical seed rules to the both coding and non coding areas. Additional algorithms applied were rna22 and miRWalk 2.0 that are based on the principles of cystolic miRNA interactions. The nuclear encoded genome/MitomiR interaction was explored with miRDB. Gene targets were filtered with MitoMiner4.0 for mitochondrial proteins with an Integrated Mitochondrial Protein Index (IMPI) > 0.7. Identified mitochondrial protein was then characterized in Uniprot and Kyoto Encyclopedia of Genes and Genomes (KEGG) for biological function. If an open reading frame (ORF) protein was identified, putative biological role was explored in the Protein Data Base (PDB), Protein Atlas and HGNC Comparison of Orthology Predictions (HCOP). 2D and 3D modelling of ORF protein was performed using Raptor-X and I-Tasser respectively, to further characterize ORF protein function. URL shortcut to databases and algorithms used are listed.

Archival data, from our previous work that identified 16 miRNA to have differential expression in DCD PNF^6^, was re-analyzed using a number of publically available computational algorithms. Figure 1 summarizes computational algorithms, databases and strategy applied. The archival data analysed in this present manuscript originated from RNA that had been isolated from post perfusion formalin fixed paraffin embedded (FFPE) trucut liver biopsies using High Pure FFPE RNA micro kit (Roche Diagnostics Ltd, Hertfordshire, UK). The three DCD groups compared were 1. PNF retransplanted within a week (n = 7), 2. good functional outcome (n = 7) peak aspartate transaminase (AST) ≤ 1000 IU/L and 3. early graft dysfunction (n = 9) peak AST ≥ 2500 IU/L. High throughput expression analysis was performed using Affymetrix GeneChip miRNA 2.0 Arrays according to manufacturer recommendations.

Initial data analysis and mining was performed using Qlucore Omics Explorer (QOEv2.1) version 2.1. Statistical significance level was set at p = 0.05 with a false discovery rate q value of 0.95^6^. The DCD PNF miRNA selected for further in silico analysis for this present manuscript are summarized in Table 1. Only miRNA with human homology has been considered. miRBase v21 published miRNA sequences and annotation have been adhered to^7–9^.

**Table 1:** Summary of Primary Non Function microRNA selected for in silico analysis. Mature microRNA (miRNA) identifiers have been used from miRBase. Table summarizes miRNA array fold change, accession number and sequence, total number of miRNA gene targets as identified by miRDB v21, total number of miRNA gene targets recorded in MitoMiner4.0 and number of MitoMiner4.0 identified genes with an Integrated Mitochondrial Protein Index (IMPI) score >0.7. miRNA that have been experimentally isolated from mitochondria is marked “yes”(Y) to identify as a MitomiR.

miRNA	Fold Change (p < 0.05)	Accession Nos Sequence	miRDB	MitoMiner	IMPI > 0.7	MitomiR
miR-107	−1.8 (p = 0.02)	MI0000114 50-agcagcauuguacagggcuauca-72	433	29	1	Y^5,55^
miR-378	−1.6 (p = 0.04)	MIMAT0000731 5-cuccugacuccagguccugugu-26	169	17	0	Y^66^
miR-23b	−2.6 (p = 0.02)	MIMAT0000418 20-uggguuccuggcaugcugauuu-41	866	76	3	Y^5,66^
miR-122-5p	−6 (p = 0.02)	MIMAT0000421 15-uggagugugacaaugguguuug-36	187	23	0	Y^67^
miR-103a-3p	−2.2 (p = 0.02)	MIMAT0000101 48-agcagcauuguacagggcuauga-70	433	29	1	Y^5,68^
miR-125b-5p	−1.8 (p = 0.03)	MIMAT0000423 15-ucccugagacccuaacuuguga-36	476	21	0	Y^5,66^
miR-24	−2 (p = 0.04)	MIMAT0000080 44-uggcucaguucagcaggaacag-65	552	38	0	Y^66^
let-7a	−1.6 (p = 0.03)	MIMAT0000062 6-ugagguaguagguuguauaguu-27	435	24	0	Y^66^
miR-191-5p	−1.5 (p = 0.02)	MIMAT0000440 16-caacggaaucccaaaagcagcug-38	55	1	0	
miR-194-5p	−1.6 (p = 0.04)	MIMAT0000460 15-uguaacagcaacuccaugugga-36	291	20	1	
miR-296-3p	−1.6 (p = 0.02)	MIMAT0004679 48-gaggguuggguggaggcucucc-69	364	17	1	
miR-455-3p	−1.6 (p = 0.03)	MIMAT0004784 54-gcaguccaugggcauauacac-74	305	13	0	
miR-940	−2 (p = 0.01)	MIMAT0004983 60-aaggcagggcccccgcucccc-80	1024	75	1	
let-7d-5p	−1.5 (p = 0.03)	MIMAT0000065 8-agagguaguagguugcauaguu-29	438	25	0	Y^66^
miR-22-3p	−1.6 (p = 0.047)	MIMAT0000077 53-aagcugccaguugaagaacugu-74	430	17	0	Y^66^
miR-155-5p	−2 (p = 0.01)	MIMAT0000646 4-uuaaugcuaaucgugauaggggu-26	311	19	0	

### Liver PNF MitomiRs

To identify the miRNA that are MitomiRs i.e documented as being isolated from mitochondria, published next generation sequencing (NGS), miRNA array, RNA cross-linking immunoprecipitation (CLIP), RT-qPCR or functional data was cross referenced. The interactions of MitomiRs with the mt genome were visualized using MitoWheel^10^, a graphical summary of the circular human mt genome (16569 base pairs) that is based on the standard revised Cambridge reference sequence (GenBank NCBI database accession number NC_012920)^11,12^.

**Figure 2:**
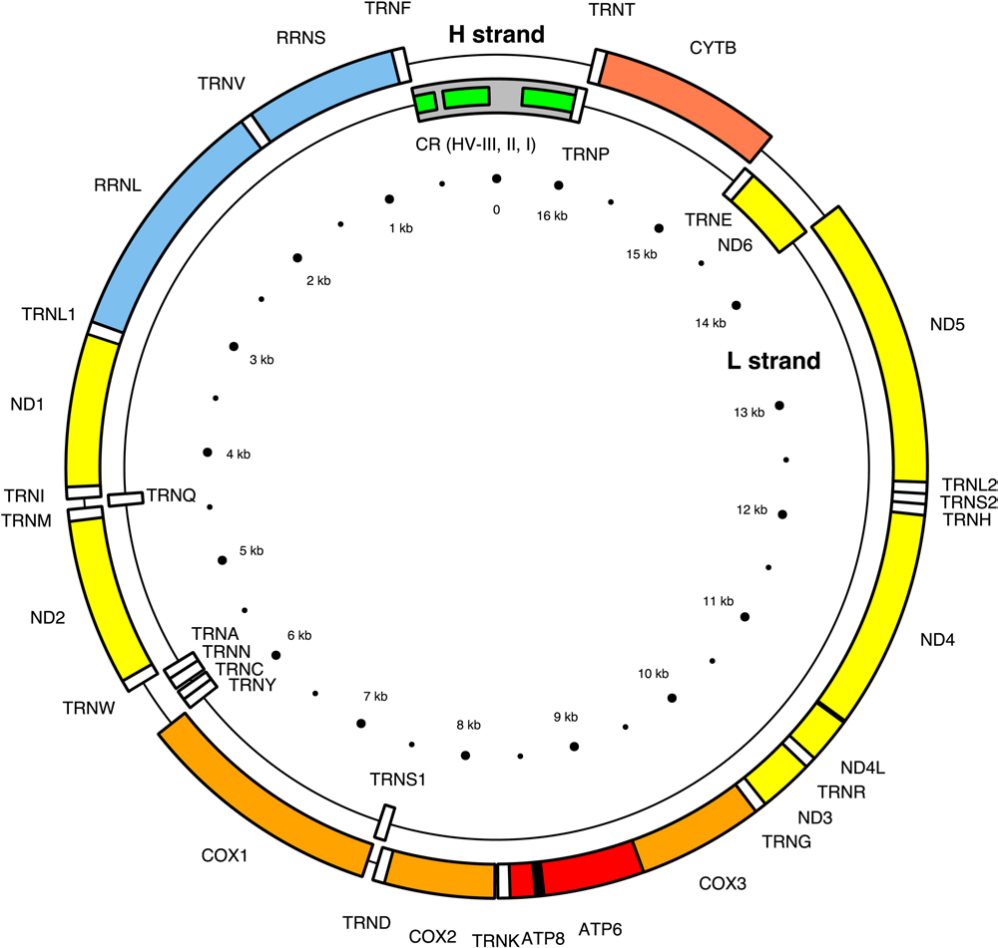
Map of the Mitochondrial Genome. The H (heavy) strand is the outer circle and L (light) strand is the inner circle. There are 22 transfer RNA (TRN) genes for the following amino acids: F, V, L1 (codon UUA/G), I, Q, M, W, A, N, C, Y, S1 (UCN), D, K, G, R, H, S2 (AGC/U), L2 (CUN), E, T and P, which are shown as white boxes. There are 2 ribosomal RNA (RRN) genes: S (small subunit, or 12S) and L (large subunit, or 16S) shown as blue boxes. There are 13 protein-coding genes: 7 for NADH dehydrogenase subunits (ND, yellow boxes), 3 for cytochrome c oxidase subunits (COX, orange boxes), 2 for ATPase subunits (ATP, red boxes), and one for cytochrome b (CYTB, coral box). Two gene overlaps are indicated (ATP8-ATP6, and ND4L-ND4, black boxes). The control region (CR) is the longest non coding sequence (grey box) and its three hypervariable regions are indicated (HV, green boxes). The most polymorphic area is HVRI (16024–16383) followed by HVRII (57–372) and HVRIII (438–574). Numbering is from the Cambridge Reference Sequence (van Oven 2009). Map of the human mitochondrial genome courtesy of Emmanuel Douzery (14).

Initial exploration of MitomiR interactions with the mt genome used simple canonical binding rules of a 6 seed sequence, as identified from nucleotide 2 to 7 from the 5′ prime end of a given miRNA. An online sequence editor^13^ was used to interconvert between RNA and DNA to allow for consensus matching of miRNA seed sequence to the input sequence of the mt genome. See Fig. 2 for map summary of the human mt genome^14^. Further in silico analysis of MitomiR interactions with the mt genome were performed using rna22 and MiRWalk 2.0 both at default settings. rna22 uses the Teiresias algorithm to predict miRNA:mRNA heteroduplexes allowing for both bulges and G:U wobbles^15,16^. While MiRWalk 2.0 uses predicted and validated information on miRNA-target data to query a given sequence for identification of miRNA species and their binding points^17,18^. Both rna22 and MiRWalk are based on the known cystolic rules of miRNA seed behaviour.

### Liver PNF miRNA and the mitochondrial proteome

In order to identify interactions of a given PNF miRNA with the mt proteome, the mature miRNA identifier was used for data mining of predicted gene targets using miRDB (MicroRNA Target Prediction And Functional Study Database)^19^. miRDB is an online database that uses a miRNA target prediction program based on a support vector machine (supervised machine learning). For target mining, threshold values were kept at the default producing generated target thresholds between 50–100, the higher the score the more confidence in a given prediction^20,21^.

The miRDB generated list of predicted gene targets for a given PNF miRNA were then exported into MitoMiner4.0^v2016 APR^(last accessed July 2017)^22,23^. MitoMiner4.0 uses data from the MitoCarta2.0 inventory of genes that encode for mitochondrial proteins originally derived from mitochondrial mass spectrometry data^2,3^. MitoMiner4.0 further integrates this data with data from Universal Protein Resource (UniProt), Gene Ontology, Online Mendelian Inheritance in Man, HomoloGene, Kyoto Encyclopaedia of Genes and Genomes (KEGG) and PubMed to calculate the Integrated Mitochondrial Protein Index (IMPI) score. The MitoMiner4.0 algorithm uses machine learning to evaluate these multiple data sources with a random forest learning classifier. Genes with an IMPI score of 0.7 or greater, being regarded as strong evidence for gene product mitochondrial localization^22,23^.

The mitochondrial proteins (IMPI > 0.7), computationally identified to be regulated by a given PNF miRNA were characterized in more detail using UniProt for protein sequence and annotation data^24^, and KEGG, to explore protein biological pathways^25,26^. If the PNF miRNA target was an open reading frame (ORF), the nucleotide sequence was translated in UniProt^24^ and the FASTA amino acid sequence downloaded for protein modelling and prediction. An ORF encodes for a hypothetical protein that presently does not have a characterized homologue in the protein database but has some data demonstrating gene product expression from microarray and mass spectrometry^27,28^. To explore ORF protein features and putative function, a search was performed via the Protein Data Bank (PDB) portal based on FASTA sequence using BLAST with the following limits of mask low complexity, e value cut off 10 and for retrieval of homologous proteins at 70% sequence identity^29,30^. For documented tissue and cell expression the Protein Atlas was searched^31,32^. For orthology exploration the HGNC Comparison of Orthology Predictions (HCOP) was used^33^. Protein secondary structure was explored with RaptorX^34,35^ and the dictionary of protein secondary structure nomenclature applied. I-Tasser (Iterative Threading ASSEmbly Refinement) that uses a hierarchial approach was used for further 3D protein structure modelling, ligand binding and enzymatic site predictions^36,37^.

## Results

### Liver PNF microRNA

Based on archival miRNA array data^6^, 16 miRNA were identified as potential distinguishers for DCD liver PNF with widespread interactions with the nuclear genome as predicted by miRDB. All of the identified DCD PNF miRNA were downregulated in expression on miRNA array analysis. Of these 16 miRNA, 10 have been identified to be MitomiRs based on published data that has demonstrated their presence in mitochondria. Whereas, MitoMiner4.0, characterized 6 PNF miRNA to be targeting transcripts that encoded for mitochondrial proteins IMPI > 0.7 (Table 1).

Considering PNF MitomiRs alone, seven had no direct regulatory action on the nuclear derived mt proteome as identified by MitoMiner4.0 (miR-378, miR-122-5p, miR-125b-5p, miR-24, let-7a, let-7d-5p, miR-22-3p). Leaving 3 MitomiRs (miR-107, miR-23b, miR-103a-3p) with a MitoMiner4.0 identified interaction with the nuclear encoded mt proteome. One PNF miRNA (miR-191-5p) was not recognized as either being a MitomiR or having any regulatory interaction with the nuclear encoded mt proteome (Table 1).

### Predicted Interactions of MitomiRs with the Mitochondrial genome

Three MitomiRs miR-107, miR-103a-3p and miR-22-3p had no identified interactions with the mt genome based on canonical seed binding predictions (Table 2). However, miR-107 and miR-103a-3p had interactions with the mitochondrial encoded nuclear genome based on target mitochondrial protein having an IMPI > 0.7 (Table 1). The remaining 7 MitomiRs (miR-378, miR-23b, miR-122-5p, miR-125b-5p, miR-24, let-7a, let-7d-5p) had multiple potential sites of interaction with the mt genome that included both coding and non coding areas (Table 2). Non coding areas for interaction included the Hypervariable Region 1 (HVRI) for miR-23b and miR-122-5p; with miR-23b, let-7a and let-7d-5p additionally interacting with the control region. The majority of the MitomiR targets were on the light strand (L-strand) (Table 2).

**Table 2:** MitomiR seed predictions for interactions with transcripts from the mitochondrial genome. Table summarizes total number of seed binding sites based on canonical binding, area of mitochondrial genome affected, its functional area and the transcript affected. Small “r” after nucleotide number means reverse and is synonymous with the heavy strand. Non coding areas are Hypervariable Region 1 (HVR1) and the Control Region (CR).

MitomiR	Seed Bind Points		Mt Gene Area	Functional Area	Transcript
	Nos	Location			
miR-107	0	—	—	—	—
miR-378	5	5590–5595	MT-TA	Coding	tRNA-Ala, position 70 in acceptor stem
		5867–5862r	MT-TY	Coding	tRNA-Tyr, position 29
		6483–6478r	MT-C01	Coding	subunit COI of complex IV (cytochrome c oxidase)
		6507–6502r	MT-C01	Coding	subunit COI of complex IV (cytochrome c oxidase)
		15264–15259r	MT-CYB	Coding	cytochrome b subunit of C3 (ubiquinol:cytochrome c oxidoreductase)
miR-23b	6	915–920	MT-RNR1	Coding	12 S ribosomal RNA
		3214–3219	MT-RNR2	Coding	16 S ribosomal RNA
		4825–4830	MT-ND2	Coding	subunit ND2 of complex I (NADH dehydrogenase)
		11307–11312	MT-ND4	Coding	subunit ND4 of complex I (NADH dehydrogenase)
		16055–16060	MT-HVR1	Non coding	
		16472–16467r	MT-CR	Non coding	
miR-122–5p	19	1233–1238	MT-RNR1	Coding	12 S ribosomal RNA
		2254–2259	MT-RNR2	Coding	16 S ribosomal RNA
		2414–2419	MT-RNR2	Coding	16 S ribosomal RNA
		4014–4019	MT-ND1	Coding	subunit ND1 of complex I (NADH dehydrogenase)
		4649–4654	MT-ND2	Coding	subunit ND2 of complex I (NADH dehydrogenase)
		4909–4914	MT-ND2	Coding	subunit ND2 of complex I (NADH dehydrogenase)
		4931–4936	MT-ND2	Coding	subunit ND2 of complex I (NADH dehydrogenase)
		5890–5895	MT-TY	Coding	tRNA-Tyr. position 2 in the acceptor stem
		8475–8480	MT-ATP8	Coding	subunit ATP8 of complex V (ATP synthase)
		8791–8796	MT-ATP6	Coding	subunit ATP6 of complex V (ATP synthase)
		9848–9853	MT-CO3	Coding	subunit COIII of complex IV (cytochrome c oxidase)
		10981–10986	MT-ND4	Coding	subunit ND4 of complex I (NADH dehydrogenase)
		11494–11499	MT-ND4	Coding	subunit ND4 of complex I (NADH dehydrogenase)
		12963–12968	MT-ND5	Coding	subunit ND5 of complex I (NADH dehydrogenase)
		13452–13457	MT-ND5	Coding	subunit ND5 of complex I (NADH dehydrogenase)
		13491–13496	MT-ND5	Coding	subunit ND5 of complex I (NADH dehydrogenase)
		15153–15148r	MT-CYB	Coding	cytochrome b subunit of C3 (ubiquinol:cytochrome c oxidoreductase)
		15268–15273	MT-CYB	Coding	cytochrome b subunit of C3 (ubiquinol:cytochrome c oxidoreductase)
		16261–16266	MT-HVR1	Non coding	
miR-103a-3p	0	—	—	—	—
miR-125b-5p	4	4816–4811r	MT-ND2	Coding	subunit ND2 of complex I (NADH dehydrogenase)
		5667–5672	MT-TN	Coding	tRNA-Asn. position 63 in the T-stem
		9595–9590r	MT-CO3	Coding	subunit COIII of complex IV (cytochrome c oxidase)
		15265–15260r	MT-CYB	Coding	cytochrome b subunit of C3 (ubiquinol:cytochrome c oxidoreductase)
miR-24	3	2478–2473r	MT-RNR2	Coding	16 S ribosomal RNA
		7227–7222r	MT-CO1	Coding	subunit COI of complex IV (cytochrome c oxidase)
		13916–13911r	MT-ND5	Coding	subunit ND5 of complex I (NADH dehydrogenase)
	13	41–46	MT-CR	Non coding	
		2735–2730r	MT-RNR2	Coding	16 S ribosomal RNA
		4221–4226	MT-ND1	Coding	subunit ND1 of complex I (NADH dehydrogenase)
		7367–7372	MT-CO1	Coding	subunit COI of complex IV (cytochrome c oxidase)
		7500–7505	MT-TS1	Coding	tRNA-SerUCN position 16 in the DHU-loop
		9320–9325	MT-CO3	Coding	subunit COIII of complex IV (cytochrome c oxidase)
		9968–9973	MT-CO3	Coding	subunit COIII of complex IV (cytochrome c oxidase)
		10211–10216	MT-ND3	Coding	subunit ND3 of complex I (NADH dehydrogenase)
		11578–11583	MT-ND4	Coding	subunit ND4 of complex I (NADH dehydrogenase)
		12591–12596	MT-ND5	Coding	subunit ND5 of complex I (NADH dehydrogenase)
		14055–14060	MT-ND5	Coding	subunit ND5 of complex I (NADH dehydrogenase)
let-7a		14377–14382	MT-ND6	Coding	subunit ND6 of complex I (NADH dehydrogenase)
let-7d-5p		15665–15670	MT-CYB	Coding	cytochrome b subunit of C3 (ubiquinol:cytochrome c oxidoreductase)
miR-22–3p	0				

Repeating the above analysis using open source computational algorithms which are based on the principles of cystolic miRNA interactions. rna22, did not identify MitomiRs miR-107, miR-378, miR-103a-3p and 125b-5p to have an interaction with the mt genome. Otherwise, the remaining MitomiRs were characterized to interact with coding areas of the mt genome. Only miR-122-5p targeted non coding areas that included the HVR1 (see Supplementary Table S1). While MiRWalk did not recognize any of the PNF MitomiRs to be interacting with the mt genome (see Supplementary Table S2). For the remainder of this in silico analysis, the initial canonical MitomiR interactions with the mt genome based on conventional seed binding as documented in Table 2 has been applied as the rules of MitomiR binding in the matrix are not established.

### Predicted PNF microRNA mitochondrial protein regulation

**Figure 3:**
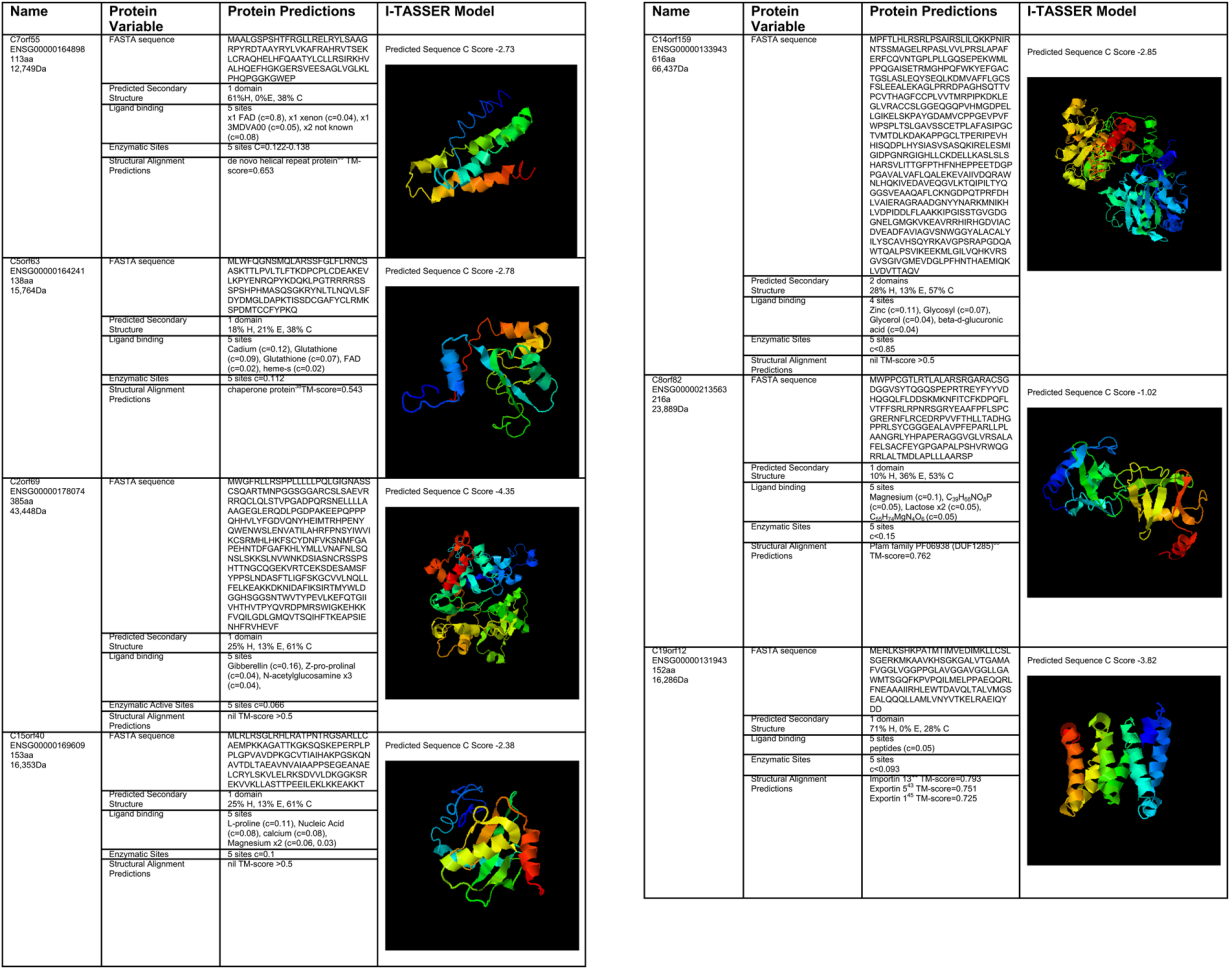
Summary of nuclear encoded mitochondrial ORF proteins targeted by Primary Non Function (PNF) miRNA. Characteristics of the ORF protein include Ensembl gene identification (ENSG), amino acid (aa) length, weight in daltons (Da) and FASTA sequence of translated protein from ORF. In all cases isoform 1 has been selected, apart from C19orf12 where isoform 4 has been used for protein modelling. Predicted secondary structure is documented using the following nomenclature: alpha helix/4 turn helix (H), extended strand in parallel and/or anti-parallel β-sheet conformation (E) and coil (C). 3D model was generated using I-TASSER, estimated global accuracy of model is in −5 to 2, C score > −1.5 indicative of a model with correct global topology. Snap shot image of 3D model generated from I-TASSER has been included. Ligand binding and enzymatic activity protein prediction data have been generated using I-TASSER, the higher the C score, the greater the confidence of the prediction, range 0–1. Structural alignment predictions of function derived from the Protein Data Bank, TM-score is a metric for measuring the structural similarity of two protein models, TM-score has the value in 0 to 1, where 1 indicates a perfect match between two structures.

Considering PNF miRNA regulation of the mt proteome, 7 Open reading frame (ORF) proteins or conserved hypothetical proteins were identified with an IMPI > 0.7. The mitochondrial ORF proteins identified were small, ranging from 113 to 616 amino acids. Orthology predictions demonstrated them to be highly conserved. All appear to localize to mitochondria, with functions that range from cofactor for ATP Synthase assembly (C7orf55), REDOX balance (C5orf63, C2orf69, C8orf82), importin/exportin ankyrin motif protein (C19orf12) or remain as an uncharacterized conserved mitochondrial protein (C15orf40, C14orf159) (Table 3 and Fig. 3). The interaction of PNF miRNA with the mt proteome appears to be with little redundancy. In that one miRNA typically targets one ORF (Table 3). However, both miR-107 and miR-103 target the C7orf55 transcript, while miR-23b has three transcript targets in the form of C5orf63, C2orf69 and C15orf40, two of which are involved in REDOX (C5orf63, C2orf69).

**Table 3:** Primary Non Function (PNF) miRNA interactions with the mitochondrial proteome. The table summarizes PNF miRNA interactions with identified transcripts of open reading frames (ORF), Ensembl gene identification (ENSG), location of ORF, number of nucleotides (nt), homolog protein product of ORF and ontological biological process associated with protein as identified by the Protein Database. miRDB target score range 50–100, the higher the score the more likely that a given miRNA targets given gene. Further details of protein class, protein evidence and subcellular localization from Protein Atlas are also associated with identified ORF protein. If ORF protein is recognized by Mitocarta2.0 to be a mitochondrial protein (IMPI > 0.7) it is marked as “yes” (Y) and any ascribed function is summarized.

PNF miRNA	Gene Target	Homolog protein	Gene Ontology Biological Process	Protein class	Subcellular localization	Evidence at Protein Level	MitoCarta
miR-107 miR-103a-3p	C7orf55	UPF0562 family FMC1 (Formation of mitochondrial complex V assembly factor 1)	negative regulation of lipid catabolic process	Intracellular Protein	Mitochondrial Matrix	Y	Y mt biogenesis
	ENSG00000164898						
	7q34						
	342nt						
	miRDB target score 61						
	IMPI 1						
miR-23b	C5orf63	Glutaredoxin-like protein	oxidation-reduction, contains phosphorylation and O-glycosylation sites	Intracellular Protein	Mitochondrial	Transcript	—
	ENSG00000164241						
	5q23.2						
	417nt						
	miRDB target score 64						
	IMPI 0.8						
	C2orf69	UPF0565 family UDP-glucose: NAD+ 6-oxidoreductase	oxidoreductases, specifically those acting on the CH-OH group of donor with NAD+ or NADP+ as acceptor.	Secreted protein	Mitochondrial Matrix	Y	Y
	ENSG00000178074						
	2q33.1						
	1158nt						
	miRDB target score 99						
	IMPI 0.72						
	C15orf40	UPF0235 family	uncharacterized	Intracellular Protein	Mito and Nucleus	Y	—
	ENSG00000169609						
	15q25.2						
	462nt						
	miRDB target score 55						
	IMPI 0.79						
miR-194–5p	C14orf159	UPF0317 family	uncharacterized	Intracellular Protein	Mitochondrial Matrix	UPF0317 family	—
	ENSG00000133943						
	14q32.11						
	1851nt						
	miRDB target score 61						
	IMPI 1						
miR-296–3p	C8orf82	UPF0598 family	Redox signal transduction	Intracellular Protein	Mitochondrial Matrix and Nucleus	Y	Y mt biogenesis
	ENSG00000213563						
	8q24.3						
	651nt						
	miRDB target score 69						
	IMPI 0.72						
miR-940	C19orf12	Family not named Ankyrin motif	In response to oxidative stress, relocates to the cytosol forming aggregates that partially co-localize with mitochondria, apoptosis, calcium balance role, lipid metabolism.	Plasma membrane	Mitochondrial and endoplasmic reticulum	Y	—
	ENSG00000131943						
	19q12						
	459nt						
	mIRDB target score 72						
	IMPI 1

Based on homology, C5orf63 protein is a small glutaredoxin-like protein with some structural alignment to a chaperone protein^38^ with predicted ligand binding to glutathione, FAD and heme-s consistent with a role in REDOX (Fig. 3). The evidence for C2orf69 protein a hypothetical oxidoreductase is stronger, with supporting data at protein level and in MitoCarta2.0 (Table 3). Both of these REDOX ORF proteins are identified to have mitochondrial localization. Another mitochondrial ORF protein with a role in REDOX, is C8orf82 protein that is regulated by miR-296-5p, it has both protein and Mitocarta2.0 supporting evidence. From homolog functional data it has pleckstrin homology for nucleotide ligand binding with a proposed role for signal transduction during oxidative stress^39^.

C7orf55 protein, also known as Formation of Mitochondrial Complex 5 Assembly Factor 1 homolog (FMC1), has a documented role in mitochondrial biogenesis according to MitoCarta2.0 and supporting protein expression evidence. Both miR-107 and miR-103a-3p appear to be involved in FMC1 translational control. Putatively, FMC1 is a matrix localizing protein that is needed for Complex 5 (ATP Synthase) stability and organization at high temperatures. When there is no FMC1 available at times of cell stress, the subunits of ATP Synthase are unable to make a functional oligomer and aggregate in the matrix^40^. FMC1 has been identified to belonging to the family of proteins carrying the LYR motif (pfam13233: Complex1_LYR_2) that contributes to Fe-S cluster biogenesis (Table 3 and Fig. 3).

Out of the 7 PNF ORF proteins identified, C19orf12 protein appears to be the most well studied but within the clinical context of neurological disease. It has 9 splice versions generating a protein of variable size (range 68–152 amino acids). The protein has been identified to be a component of the endoplasmic reticulum and the mitochondrial associated membrane. The C19orf12 protein co-localizes in mitochondria under oxidative stress, where it supports the transfer of lipids and Ca^2+^ ions to promote autophagy^41,42^. However, it is not recognized to be a mitochondrial protein by MitoCarta2.0 but there is some reported evidence for mitochondrial protein expression based on tissue analysis^32^. 3D modelling predictions for C19orf12 protein (isoform 4) give structural alignment predictions that identify it be a putative importin/exportin^43,44^. This importin/expotin has predicted ability to bind basic residues such as nucleic acids (Fig. 3).

## Discussion

There is a lack of data on predictive markers in DCD liver transplantation and this has led to the re-analysis of archival miRNA array data, despite its limitations and prior demonstration that miRNA is a poor discriminator of functional outcome^6^. However, by focusing on mitochondrial biology, this in silico work presents an argument for how PNF miRNA interacts with the mt genome and nuclear encoded mt proteome to form the mitochondrial events that culminate in PNF. Using this strategy a number of novel/hypothetical ORF proteins have been identified, as well as giving insight into MitomiR behaviour in the context of PNF. Out of the 16 PNF miRNAs identified, 10 can be classified as MitomiRs as they have previously isolated from mitochondria (Table 1). Four of these MitomiRs have been functionally characterized (miR-378, miR-24, miR-23b and let-7a) with roles in control of hypoxia induced apoptosis^45^, systemic energy homeostasis^46^, oxidative capacity^47^, insulin signalling^48^ and ROS generation^49^. All of these processes are of importance for good graft function and survival in liver transplantation.

The mt genome is compact, with little or no intergenic non coding sequences. Additionally, mitochondrial messenger RNA lack a non translated leader and trailing sequence. Other unique features of the mt genome are the control region and the hypervariability region (HVR). The control region contains the point where transition from RNA to DNA occurs, thereby being the point of control for both replication of the mt genome and mitochondrial transcription (Fig. 1). While HVR, despite its name, encodes for a RNA transcript that has a conserved secondary structure^50^. These features of the mt genome, combined with canonical seed predictions, identify that the majority of the PNF MitomiRs are interacting with coding areas of the mt genome. While non coding area interactions are limited to miR-23b and miR-122-5p for the HVR1, and miR-23b, let-7a and let-7d-5p interacting with the control region. Additionally, the majority of the MitomiR interactions in the cellular context of PNF appear to be directed against the L-strand, suggesting that in PNF there is a loss of mitochondrial polycistronic balance (see Table 2).

In silico, MitomiRs miR-107, miR-103a-3p and miR-22-3p do not have any identified interactions with the transcribed mt genome. Suggesting that unconventional seed interactions, both non canonical and alternative miRNA seed sites are of greater importance in the mitochondrial matrix than the accepted miRNA regulatory events of the cytosol^51^. Some of these atypical seed rules have already been described for miR-107 and 103a-3p in the cytosol, which target mRNA ORFs and the 5′ UTR respectively^52,53^.

The majority of MitomiRs are derived from the nuclear genome with a few from the mt genome (miR-4485-3p, miR-1973, miR-4284)^4,54,55^. There is also some evidence that small noncoding RNA (20-40 nucleotides) derived from the mt genome stay within mitochondria and promote transcription^56^. Whereas nuclear generated MitomiRs traffic between all compartments of the cell and human body to act as translational repressors^57,58^. In silico, the PNF MitomiRs (miR-107, miR-103a-3p, miR-22-3p) that do not have any identified interactions with the mt genome based on canonical seed binding rules, do however interact with the nuclear encoded mitochondrial proteome. Potentially, this distribution and differential activity of MitomiRs in the nucleus and mitochondria could provide a mechanism of anterograde retrograde nuclear mitochondrial communications that contribute to mitochondrial dysfunction and failure in this clinical model of liver transplantation^59^.

Most miRNA computational algorithms are a combination of validated knowledge bases and predicted canonical base pairing of the miRNA seed sequence with mRNA in the context of the cytosol^16,18,51^. Nevertheless, canonical predictions limited to the 3′UTR can be an over simplification of events as RNA binding protein immunoprecipitation studies have demonstrated that miRNA can bind both non canonically and to sites outside the 3′UTR. In plants, it is established that miRNA binding to coding regions is more common, with binding to the 5′UTR (promoter and enhancer area of a gene) leading to up regulation. Whereas in mammals, miRNA has mainly been characterized to bind to the 3′UTR^51,60^. Additionally, the mt genome is highly compact. Therefore, MitomiR seed binding rules and behaviour in the matrix are likely to be different to that of the cytosol. MitomiR binding events and regulatory effect remain unknown and need further characterization, making it presently difficult to categorically state in silico, whether a given MitomiR will be a translational repressor or enhancer in the present model^59^.

Mitochondria are a major source of ROS and maintenance of REDOX balance is essential, in order to protect from oxidative damage, and its cellular sequelae. While previous work has associated poor outcome in liver transplantation with low levels of oxidant enzymes^1^. The mitochondrial ORF proteins, whose translation in silico that have been identified to be regulated by PNF miRNA (Table 3), have predicted functional roles that appear to be biologically plausible and relevant to the clinical model of PNF in liver transplantation. C5orf63 and C2orf69 proteins are both characterized to be oxidoreductases. While C8orf82 protein act as a signal transducer during oxidative stress^39^.

C7orf55 protein, or FMC1 identified homolog is involved in the formation and stability of ATP synthase and thereby bioenergetic mitochondrial efficiency. ATP synthase is the concluding protein complex of OXPHOS and is needed to drive the proton gradient for ATP generation^40,61^. Graft recovery and viability is intimately involved with the ability of mitochondria to generate high energy phosphates^62^ and an inadequate amount of FMC1 protein would be predicted to lead to ATP synthase failure. In contrast, C19orf12 protein, with an ankyrin motif, appears to act as a shuttle between the cytosol and mitochondria during oxidative stress^41,42^. Structural based assignment identify the protein to be an importin/exportin chaperone^43,44,63^ which could be involved in the control of nucleic acid traffic between the nuclear, cytosolic and mitochondrial compartments to define cell viability^64,65^.

There is presently no biological data on the in silico identified protein targets and the MitomiRs computationally characterized are yet to be associated with their gene targets. The original experiments^6^ were based on the DCD liver, which is subjected to a warm ischemic period that is one of the determinants of this graft’s unpredictable behavior on reperfusion. However, in order to validate and understand whether the identified PNF MitomiRs and ORF proteins are biologically true and relevant to liver transplantation, our future work will focus on their expression profile in different donor liver types (neonatal, living related, DCD and Donor after Brainstem Death), combined with *in vitro* experiments to characterize their interactions with the mitochondrial proteome.

In conclusion, the behaviour of MitomiRs within the mitochondrial matrix are ill understood and more study is needed. However, the emerging data on their compartmentalized functional roles could explain, how a cell sets and adjusts its mitochondrial proteome according to cellular energy needs and stresses. Additionally, a number of small mitochondrial ORF proteins that could be of relevance in liver transplantation haven been identified. Despite the limitations of this computational work, validation of these in silico observations on MitomiRs and ORF proteins could provide a way to understand the limits of a DCD liver in transplantation.

## Electronic supplementary material

Supplementary Material
