# Supplementary material for: An in silico argument for mitochondrial microRNA as a determinant of primary non function in liver transplantation
Source: Sci Rep. 2018 Feb 15;8:3105. doi: 10.1038/s41598-018-21091-9 (PMC5814406; doi:10.1038/s41598-018-21091-9)
Supplement: Supplementary file 1 — Supplementary Material [file 41598_2018_21091_MOESM1_ESM.pdf]

Supplementary Material S1 – S2

An in silico argument for mitochondrial microRNA as a determinant of primary non function in liver transplantation.

Shirin Elizabeth Khorsandi, Siamak Salehi, Miriam Cortes, Hector Vilca-Melendez, Krishna Menon, Parthi Srinivasan, Andreas Prachalias, Wayel Jassem, Nigel Heaton

S1 RNA22 predictions of Primary Non Function (PNF) mitochondrial miRNA (MitomiR) interactions with the Mitochondrial Genome (mt genome). Table summarizing MitomiR interaction with the mt genome predicted by RNA22 that include location and function of site of interaction, the miRNA:mRNA heteroduplexes and its probability.

<table>
<tr><th>miR Name</th><th>mtgenome</th><th>leftmost position of predicted target site</th><th>folding energy (in -Kcal/mol)</th><th>heteroduplex</th><th>p value</th></tr>
<tr><td>miR-let-7d-5p</td><td>MT-RNR1 coding for the 12S ribosomal RNA</td><td>1223</td><td>-16.70</td><td>CCCCGATCAACCTCACCACCTCT
|||||| || ||||||
TTGATACGTTGGA-TGATGGAGA</td><td>4.2E-2</td></tr>
<tr><td>miR-23b</td><td>MT-RNR2 coding for the 16S ribosomal RNA.</td><td>1675</td><td>-12.10</td><td>AACCTAGC--CCCA--AACCCA
:||| ||| ||||||
TTTAGTCGTACGGTCCTTGGGT</td><td>7.31E-2</td></tr>
<tr><td>miR-122-5p</td><td>MT-RNR2 coding for the 16S ribosomal RNA.</td><td>1678</td><td>-13.20</td><td>CTAGCCCCA-AACC-CACTCCA
|:| ||| | ||||||
GTTTGTGGTAACAGTGTGAGGT</td><td>7.31E-2</td></tr>
<tr><td>miR-23b</td><td>MT-RNR2 coding for the 16S ribosomal RNA.</td><td>2458</td><td>-13.20</td><td>AAAAAAGTA--AAAGGAACTCG
||| ||:| |||||:|:
TTTAGTCGTACGGTCCTTGGGT</td><td>1.67E-1</td></tr>
<tr><td>miR-23b</td><td>MT-RNR2 coding for the 16S ribosomal RNA.</td><td>2820</td><td>-15.20</td><td>ACCTCGG--AGC--AGAACCCA
||:| || ||||||
TTTAGTCGTACGGTCCTTGGGT</td><td>3.66E-1</td></tr>
<tr><td>miR-24</td><td>MT-RNR2 coding for the 16S ribosomal RNA.</td><td>3051</td><td>-15.10</td><td>AAAGTCCTACGTGATCTGAGTTC
|||| | ||| ||||::
GACAAGGACG-ACTTGACTCGGT</td><td>2.35E-1</td></tr>
<tr><td>miR-122-5p</td><td>MT-ND1 coding for subunit ND1 of complex I (NADH dehydrogenase). First nucleotide of codon 2 (CCC) for proline.</td><td>3310</td><td>-12.20</td><td>CCATGGCCAACCTCCTACTCCT
:||| || :||||
GTTTGTGGTAACAGTGTGAGGT</td><td>6.11E-2</td></tr>
<tr><td>miR-23b</td><td>MT-ND1 coding for subunit ND1 of complex I (NADH dehydrogenase). Third nucleotide of codon 188 (TCC) for serine.</td><td>3870</td><td>-15.90</td><td>ACACTAGCAGAGACCAACCGAACCCC
:|||| | ||| ||||||
TTTAGTCGT-AC-GGT--CCTTGGGT</td><td>3.39E-1</td></tr>
<tr><td>miR-122-5p</td><td>MT-ND1 coding for subunit ND1 of complex I (NADH dehydrogenase). Third nucleotide of codon 243 (CTA) for leucine.</td><td>4035</td><td>-16.70</td><td>GGAACAACATATGACGCACTCTC
:|||| ||| || |:||||:
GTTTGTGGTA-ACAGTGTGAGGT</td><td>9.05E-2</td></tr>
<tr><td>miR-23b</td><td>MT-ND2 coding for subunit ND2 of complex I (NADH dehydrogenase). Second nucleotide of the alternative start codon ATT.</td><td>4471</td><td>-12.30</td><td>TAATCCCCTGGCC--CAACCCG
|||| ||| |||||:
TTTAGTCGTACGGTCCTTGGGT</td><td>3.56E-2</td></tr>
<tr><td>miR-122-5p</td><td>MT-ND2 coding for subunit ND2 of complex I (NADH dehydrogenase). Second nucleotide of codon 176 (CGC) for arginine.</td><td>4996</td><td>-13.20</td><td>CAAA-ATC-TTAGCATACTCCT
|||| |:| ||:|||||
GTTTGTGGTAACAGTGTGAGGT</td><td>1.71E-2</td></tr>
<tr><td>miR-let-7a</td><td>Gene MT-ND2 coding for subunit ND2 of complex I (NADH dehydrogenase). First nucleotide of codon 286 (ACC) for threonine.</td><td>5325</td><td>-14.20</td><td>CCCTCCTTAACCT-CTACTTCT
:||||| ||||:||
TTGATATGTTGGATGATGGAGT</td><td>1.8E-2</td></tr>
<tr><td>miR-let-7d-5p</td><td>Gene MT-ND2 coding for subunit ND2 of complex I (NADH dehydrogenase). First nucleotide of codon 286 (ACC) for threonine.</td><td>5325</td><td>-15.80</td><td>CCCTCCTTAACCT-CTACTTCT
:||||| ||||:|||
TTGATACGTTGGATGATGGAGA</td><td>1.8E-2</td></tr>
<tr><td>miR-let-7a</td><td>Gene MT-ND2 coding for subunit ND2 of complex I (NADH dehydrogenase). Third nucleotide of codon 293 (TAC) for tyrosine.</td><td>5348</td><td>-12.40</td><td>CTACGCCTAATCTACTCCACCTCA
:||||| ||||||
TTGATATGTTGGATGA--TGGAGT</td><td>4.03E-2</td></tr>
<tr><td>miR-let-7d-5p</td><td>Gene MT-ND2 coding for subunit ND2 of complex I (NADH dehydrogenase). Second nucleotide of codon 332 (CTT) for leucine.</td><td>5464</td><td>-13.60</td><td>TACCACGCTACTCCTACCTATCTCC
|| ||||| ||:|||
TTGATACG--TTGGATG-ATGGAGA</td><td>7.42E-2</td></tr>
<tr><td>miR-let-7a</td><td>Gene MT-ND2 coding for subunit ND2 of complex I (NADH dehydrogenase). Second nucleotide of codon 333 (ACC) for threonine.</td><td>5467</td><td>-13.60</td><td>CACGCTAC-TCCTACCTATCTCC
||| ||||| ||:|||
TTGATATGTTGGATG-ATGGAGT</td><td>7.42E-2</td></tr>
<tr><td>miR-let-7a</td><td>MT-TY coding for tRNA-Tyr. Position 27 in the anticodon stem.</td><td>5869</td><td>-17.10</td><td>TGCTTCACTCAGCCATTTTACCTCA
:|| ||:|| :|||||||
TTGA--TATGTTGG-ATGATGGAGT</td><td>2.39E-1</td></tr>
<tr><td>miR-let-7d-5p</td><td>MT-TY coding for tRNA-Tyr. Position 27 in the anticodon stem.</td><td>5869</td><td>-14.80</td><td>TGCTTCACTCAGCCATTTTACCTCA
:|| ||:|| :||||||
TTGA--TACGTTGG-ATGATGGAGA</td><td>2.39E-1</td></tr>
</table>

<table>
<tr><th>miR Name</th><th>mtgenome</th><th>leftmost position of predicted target site</th><th>folding energy (in - Kcal/mol)</th><th>heteroduplex</th><th>p value</th></tr>
<tr><td>miR-122-5p</td><td>Gene MT-CO1 coding for subunit COI of complex IV (cytochrome c oxidase). First nucleotide of codon 321 (TTT) for phenylalanine.</td><td>6864</td><td>-14.19</td><td>TTAGCTGACTCGCCACACTCCA
 |:| ||||||||
GTTTGTGGTAACAGTGTGAGGT</td><td>8.91E-2</td></tr>
<tr><td>miR-122-5p</td><td>Gene MT-CO1 coding for subunit COI of complex IV (cytochrome c oxidase). First nucleotide of codon 408 (ACC) for threonine.</td><td>7125</td><td>-13.90</td><td>CCTACGCCAAAATC-CATTTCA
 ||:||| || ||:|:||
GTTTGTGGTAACAGTGTGAGGT</td><td>7.42E-2</td></tr>
<tr><td>miR-let-7a</td><td>Gene MT-CO2 coding for subunit COII of complex IV (cytochrome c oxidase). First nucleotide of codon 117 (ATC) for isoleucine.</td><td>7934</td><td>-12.20</td><td>TCTTCAAC-TCCTACATACTTCC
   || ||||| |||:||
TTGATATGTTGGATG-ATGGAGT</td><td>1.06E-1</td></tr>
<tr><td>miR-let-7d-5p</td><td>MT-TK coding for tRNA-Lys. Position 44 in the variable loop.</td><td>8333</td><td>-13.40</td><td>GATTAAGAGAACCAAC-ACCTCT
 |:|| | |||| || ||||||
TTGATAC-GTTGGATGATGGAGA</td><td>1.26E-1</td></tr>
<tr><td>miR-let-7a</td><td>MT-ATP8 coding for subunit ATP8 of complex V (ATP synthase). First nucleotide of codon 30 (AAC) for asparagine</td><td>8453</td><td>-14.70</td><td>ACACAAACTACCACCTACCTCC
   || ||| ||||||
TTGATATGTTGGATGATGGAGT</td><td>2.36E-3</td></tr>
<tr><td>miR-23b</td><td>MT-ATP6 coding for subunit ATP6 of complex V (ATP synthase). First nucleotide of codon 120 (AAG) for lysineNC_012920.1</td><td>8884</td><td>-15.70</td><td>AGATTAAAAATGCC-CTAGCCCA
 :||:| ||||| |:||||
TTTAGT-CGTACGGTCCTTGGGT</td><td>6.58E-2</td></tr>
<tr><td>miR-122-5p</td><td>MT-CO3 coding for subunit COIII of complex IV (cytochrome c oxidase). First nucleotide of codon 98 (TTC) for phenylalanine.</td><td>9498</td><td>-12.60</td><td>TCTGAGCC--TTTTACCACTCCA
   :|| |:|| ||||||
GTTTGTGGTAACAGTG-TGAGGT</td><td>3.71E-1</td></tr>
<tr><td>miR-122-5p</td><td>MT-CO3 coding for subunit COIII of complex IV (cytochrome c oxidase). Third nucleotide of codon 124 (CTA) for leucine.</td><td>9578</td><td>-13.30</td><td>AATCCCCTAGAAGTCCCACTCCT
   |:| ||| ||||||
GTTTGTGGT-AACAGTGTGAGGT</td><td>4.91E-2</td></tr>
<tr><td>miR-let-7a</td><td>MT-CO3 coding for subunit COIII of complex IV (cytochrome c oxidase). Second nucleotide of codon 212 (TCA) for serine.</td><td>9841</td><td>-12.94</td><td>AACTTTCCTCACTATCTGCTTCA
|||| ||| ||:|:|||
TTGATATGTTGGAT-GATGGAGT</td><td>2.05E-1</td></tr>
<tr><td>miR-let-7a</td><td>MT-ND4L coding for subunit ND4L of complex I (NADH dehydrogenase). First nucleotide of codon 9 (ATA) for methionine.</td><td>10494</td><td>-12.50</td><td>TACTA-GCA-TTTACCATCTCA
 |||| :|| ::||| |:||||
TTGATATGTTGGATGATGGAGT</td><td>1.59E-1</td></tr>
<tr><td>miR-let-7a</td><td>MT-ND4L coding for subunit ND4L of complex I (NADH dehydrogenase). First nucleotide of codon 20 (CTA) for leucine.</td><td>10527</td><td>-14.50</td><td>TAGTATATCGCTCAC-ACCTCA
 | ||||: :| || ||||||
TTGATATGTTGGATGATGGAGT</td><td>2.11E-1</td></tr>
<tr><td>miR-122-5p</td><td>MT-ND4 coding for subunit ND4 of complex I (NADH dehydrogenase). First nucleotide of codon 143 (CTG) for leucine.</td><td>11186</td><td>-13.30</td><td>TGAACGC-AGGCACATACTTCC
 :|||:| ||:|||:|
GTTTGTGGTAACAGTGTGAGGT</td><td>1.52E-1</td></tr>
<tr><td>miR-22-3p</td><td>MT-ND4 coding for subunit ND4 of complex I (NADH dehydrogenase). Third nucleotide of codon 232 (GCC) for alanine.</td><td>11455</td><td>-21.40</td><td>GCAGTACTCTTAAAACTAGGCGGCTA
:|||| |||| |||| |||:|||
TGTCA--AGAA-GTTGA-CCGTCGAA</td><td>7.91E-3</td></tr>
<tr><td>miR-let-7a</td><td>MT-ND4 coding for subunit ND4 of complex I (NADH dehydrogenase). Third nucleotide of codon 239 (GGC) for glycine.</td><td>11476</td><td>-13.50</td><td>GGCTATGGTATAATAC-GCCTCA
::||||: ||| :|||||
TTGATAT-GTTGGATGATGGAGT</td><td>7.91E-3</td></tr>
<tr><td>miR-122-5p</td><td>MT-ND4 coding for subunit ND4 of complex I (NADH dehydrogenase). Second nucleotide of codon 438 (TTC) for phenylalanine.</td><td>12072</td><td>-12.24</td><td>CATACACCTATCCCCCATTCTC
|| ||||| ||:||:
GTTTGTGGTAACAGTGTGAGGT</td><td>2.49E-2</td></tr>
<tr><td>miR-let-7a</td><td>MT-ND5 coding for subunit ND5 of complex I (NADH dehydrogenase). First nucleotide of codon 361 (GGA) for glycine.</td><td>13417</td><td>-14.20</td><td>GACTACTCAAAACC--ATACCTCT
:|||| || ||| |||||
TTGATATGT--TGGATGATGGAGT</td><td>1.07E-1</td></tr>
<tr><td>miR-let-7d-5p</td><td>MT-ND5 coding for subunit ND5 of complex I (NADH dehydrogenase). First nucleotide of codon 361 (GGA) for glycine.</td><td>13417</td><td>-15.70</td><td>GACTACTCAAAACC--ATACCTCT
:|||| |||| ||||||
TTGAT--ACGTTGGATGATGGAGA</td><td>1.07E-1</td></tr>
<tr><td>miR-122-5p</td><td>MT-ND5 coding for subunit ND5 of complex I (NADH dehydrogenase). Second nucleotide of codon 363 (CTC) for leucine.</td><td>13424</td><td>-15.40</td><td>CAAA-ACCATACCTCTCACTTCA
|||| ||||| || ||||:||
GTTTGTGGTA-ACAGTGTGAGGT</td><td>1.07E-1</td></tr>
<tr><td>miR-let-7a</td><td>MT-ND5 coding for subunit ND5 of complex I (NADH dehydrogenase). First nucleotide of codon 497 (GGA)</td><td>13825</td><td>-17.70</td><td>GACTTCTAACAGCCCTAGACCTCA
:||| |||:|| ||||||</td><td>6.02E-2</td></tr>
</table>

S2 MiRWalk 2.0 predictions of miRNA interactions with the mitochondrial genome.

Table summarizes location, function and EntrezID of area of interaction, the identified miRNA, MIMATid, the seed length and the probability of the interaction.

[illegible]e	EntrezID	miRNA	MIMATid	Startpos	Seed length	Seed start	Seed end	pvalue
[illegible]re TRNF	0	hsa-miR-6844	MIMAT0027589		12	368	357	0.001
Before TRNF	0	hsa-miR-6855-5p	MIMAT0027610		11	562	552	0.0039
Before TRNF	0	hsa-miR-4728-5p	MIMAT0019849		10	464	455	0.0157
RNR1	4549	hsa-miR-3201	MIMAT0015086		11	1192	1182	0.0039
RNR1	4549	hsa-miR-522-5p	MIMAT0005451		11	1199	1189	0.0039
RNR1	4549	hsa-miR-518d-5p	MIMAT0005456		11	1199	1189	0.0039
RNR1	4549	hsa-miR-523-5p	MIMAT0005449		11	1199	1189	0.0039
RNR1	4549	hsa-miR-518e-5p	MIMAT0005450		11	1199	1189	0.0039
RNR1	4549	hsa-miR-526a	MIMAT0002845		11	1199	1189	0.0039
RNR1	4549	hsa-miR-518f-5p	MIMAT0002841		11	1199	1189	0.0039
RNR1	4549	hsa-miR-7110-5p	MIMAT0028117		11	807	797	0.0039
RNR1	4549	hsa-miR-519a-5p	MIMAT0005452		11	1199	1189	0.0039
RNR1	4549	hsa-miR-519b-5p	MIMAT0005454		11	1199	1189	0.0039
RNR1	4549	hsa-miR-519c-5p	MIMAT0002831		11	1199	1189	0.0039
RNR1	4549	hsa-miR-520c-5p	MIMAT0005455		11	1199	1189	0.0039
RNR1	4549	hsa-miR-6852-5p	MIMAT0027604		10	874	865	0.0157
RNR1	4549	hsa-miR-7160-3p	MIMAT0028231		10	1469	1460	0.0157
RNR2	4550	hsa-miR-4441	MIMAT0018959		10	3124	3115	0.0157
RNR2	4550	hsa-miR-524-3p	MIMAT0002850		10	3168	3159	0.0157
RNR2	4550	hsa-miR-525-3p	MIMAT0002839		10	3168	3159	0.0157
RNR2	4550	hsa-miR-6086	MIMAT0023711		10	2843	2834	0.0157
RNR2	4550	hsa-miR-6860	MIMAT0027622		10	2552	2543	0.0157
RNR2	4550	hsa-miR-2682-3p	MIMAT0013518		10	2700	2691	0.0157
RNR2	4550	hsa-miR-410-5p	MIMAT0026558		10	1716	1707	0.0157
ND1	4535	hsa-miR-3187-3p	MIMAT0015069		11	3320	3310	0.0039
ND1	4535	hsa-miR-6507-5p	MIMAT0025470		10	3978	3969	0.0157
TRNQ	4572	hsa-miR-2392	MIMAT0019043		10	4400	4391	0.0157
ND2	4536	hsa-miR-3134	MIMAT0015000		10	4956	4947	0.0157
ND2	4536	hsa-miR-4640-5p	MIMAT0019699		10	4486	4477	0.0157
ND2	4536	hsa-miR-4709-3p	MIMAT0019812		10	4699	4690	0.0157
TRNW	4578	hsa-miR-891b	MIMAT0004913		10	5565	5556	0.0157
TRNA	4553	hsa-miR-27a-5p	MIMAT0004501		11	5654	5644	0.0039
TRNC	4511	hsa-miR-6784-5p	MIMAT0027468		10	5770	5761	0.0157
COX1	4512	hsa-miR-5094	MIMAT0021086		12	7093	7082	0.001
COX1	4512	hsa-miR-1279	MIMAT0005937		12	6900	6889	0.001
COX1	4512	hsa-miR-656-5p	MIMAT0026627		10	6043	6034	0.0157
COX1	4512	hsa-miR-4434	MIMAT0018950		10	6493	6484	0.0157
COX2	4513	hsa-miR-6757-5p	MIMAT0027414		13	7828	7816	0.0002
COX2	4513	hsa-miR-4482-3p	MIMAT0020958		10	7772	7763	0.0157
COX2	4513	hsa-miR-6785-5p	MIMAT0027470		10	7822	7813	0.0157
Between COX2 and	0	hsa-miR-6835-5p	MIMAT0027570		10	8286	8277	0.0157
TRNK	4566	hsa-miR-155-5p	MIMAT0000646		10	8319	8310	0.0157
TRNK	4566	hsa-miR-597-3p	MIMAT0026619		10	8347	8338	0.0157
ATP6	4508	hsa-miR-3936	MIMAT0018351		11	8938	8928	0.0039
ATP6	4508	hsa-miR-6069	MIMAT0023694		10	8905	8896	0.0157
COX3	4514	hsa-miR-4430	MIMAT0018945		11	9523	9513	0.0039
COX3	4514	hsa-miR-6514-5p	MIMAT0025484		11	9326	9316	0.0039
COX3	4514	hsa-miR-3609	MIMAT0017986		10	9906	9897	0.0157
ND3	4537	hsa-miR-4698	MIMAT0019793		10	10127	10118	0.0157
ND4L	4539	hsa-miR-4461	MIMAT0018983		23	10712	10690	0
ND4L	4539	hsa-miR-384	MIMAT0001075		10	10525	10516	0.0157
ND4	4538	hsa-miR-580-3p	MIMAT0003245		10	11511	11502	0.0157
TRNH	4564	hsa-miR-6819-3p	MIMAT0027539		10	12189	12180	0.0157
ND5	4540	hsa-miR-4463	MIMAT0018987		17	13067	13051	0
ND5	4540	hsa-miR-190a-5p	MIMAT0000458		11	13542	13532	0.0039
ND5	4540	hsa-miR-190b	MIMAT0004929		11	13542	13532	0.0039
ND5	4540	hsa-miR-6505-5p	MIMAT0025466		10	12753	12744	0.0157
ND5	4540	hsa-miR-6732-5p	MIMAT0027365		10	13134	13125	0.0157
ND5	4540	hsa-miR-1246	MIMAT0005898		10	12453	12444	0.0157
ND5	4540	hsa-miR-3165	MIMAT0015039		10	12466	12457	0.0157
ND5	4540	hsa-miR-381-5p	MIMAT0022862		10	13645	13636	0.0157
ND6	4541	hsa-miR-3115	MIMAT0014977		11	14525	14515	0.0039
ND6	4541	hsa-miR-6796-5p	MIMAT0027492		10	14627	14618	0.0157
CYTB	4519	hsa-miR-3605-5p	MIMAT0017981		13	15644	15632	0.0002
CYTB	4519	hsa-miR-4278	MIMAT0016910		10	15374	15365	0.0157
CYTB	4519	hsa-miR-5011-3p	MIMAT0021046		10	14908	14899	0.0157
CYTB	4519	hsa-miR-6869-5p	MIMAT0027638		10	14915	14906	0.0157
